# Development of a Predictive Model for Mortality in Hospitalized Patients With COVID-19

**DOI:** 10.1017/dmp.2021.8

**Published:** 2021-01-08

**Authors:** Yuanyuan Niu, Zan Zhan, Jianfeng Li, Wei Shui, Changfeng Wang, Yanli Xing, Changran Zhang

**Affiliations:** 1 Department of Respiratory Medicine, The Eastern Hospital of The First Affiliated Hospital, Sun Yat-sen University, Guangzhou, Guangdong Province, China; 2 Department of Respiratory Medicine, Huanggang Central Hospital, Huanggang, Hubei Province, China; 3 Department of Radiation Oncology, The First Affiliated Hospital of Guangzhou Medical University, Guangzhou, Guangdong Province, China

**Keywords:** COVID-19, risk factors, predictive model, in-hospital death, mortality

## Abstract

**Introduction:**

Early identification of patients with novel corona virus disease 2019 (COVID-19) who may be at high mortality risk is of great importance.

**Methods:**

In this retrospective study, we included all patients with COVID-19 at Huanggang Central Hospital from January 23 to March 5, 2020. Data on clinical characteristics and outcomes were compared between survivors and nonsurvivors. Univariable and multivariable logistic regression were used to explore risk factors associated with in-hospital death. A nomogram was established based on the risk factors selected by multivariable analysis.

**Results:**

A total of 150 patients were enrolled, including 31 nonsurvivors and 119 survivors. The multivariable logistic analysis indicated that increasing the odds of in-hospital death associated with higher Sequential Organ Failure Assessment score (odds ratio [OR], 3.077; 95% confidence interval [CI]: 1.848-5.122; *P* < 0.001), diabetes (OR, 10.474; 95% CI: 1.554-70.617; *P* = 0.016), and lactate dehydrogenase greater than 245 U/L (OR, 13.169; 95% CI: 2.934-59.105; *P* = 0.001) on admission. A nomogram was established based on the results of the multivariable analysis. The AUC of the nomogram was 0.970 (95% CI: 0.947-0.992), showing good accuracy in predicting the risk of in-hospital death.

**Conclusions:**

This finding would facilitate the early identification of patients with COVID-19 who have a high-risk for fatal outcome.

The outbreak of coronavirus disease 2019 (COVID-19), which is caused by severe acute respiratory syndrome coronavirus 2 (SARS-CoV-2), has rapidly spread globally. On March 11, the World Health Organization (WHO) declared it as a public health emergency.^1^

The clinical manifestations of COVID-19 are wide in spectrum, which range from asymptomatic or mildly symptomatic infections to severe pneumonia, acute respiratory distress syndrome (ARDS), and respiratory failure. Patients with severe COVID-19 have a substantial risk of prolonged critical illness and death. However, most previous studies of COVID-19 focused primarily on epidemiological and clinical characteristics; only limited evidence is available on risk factors for poor clinical outcomes.^2-5^

Here, we present details of the clinical manifestations, laboratory findings, imaging features, and clinical outcomes of patients with COVID-19 admitted to Huanggang Central Hospital, in Hubei. We aim to identify risk factors associated with in-hospital death and construct a clinical risk model to predict the fatal outcome of patients with COVID-19 upon admission.

## Patients and Methods

### Study Participants

Between January 23, 2020, and March 5, 2020, all patients with confirmed and probable COVID-19 in Huanggang Central Hospital were admitted to the hospital. Our study enrolled 150 consecutive inpatients with confirmed COVID-19 who had a definite outcome (discharged or died) during this period. All patients were diagnosed with COVID-19 pneumonia according to the WHO interim guidance.^6^ Huanggang Central Hospital, a tertiary hospital with 2500 beds, was a designated hospital for patients with COVID-19. The patients were mainly from Huangzhou District and surrounding towns. The ethics committee of Huanggang Central Hospital approved this study and granted a waiver of informed consent from the study participants.

### Data Collection

Data on epidemiological and demographic characteristics, underlying diseases, clinical manifestations, laboratory findings, chest computed tomography (CT) imaging, and outcomes of enrolled 150 patients with COVID-19 were obtained from electronic medical records. Test results at baseline rather than the worst value during hospitalization were used to predict clinical outcome. A team of experienced respiratory clinicians reviewed, abstracted, and cross-checked the data.

### SARS-CoV-2 RT-PCR Test

Throat-swab samples were obtained from all patients on admission and tested for the presence of SARS-CoV-2 infection by using real-time reverse transcriptase-polymerase chain reaction assays (RT-PCR) according to a previously described protocol.^7^ Throat-swab specimens were obtained for RT-PCR re-examination every 2 to 3 d. For patients with repeated PCR tests, the first date of the result was recorded if the patients had consecutive negative results, while the latest result and date were recorded for patients who had inconsistent results of the consecutive tests.

### Discharge

The criteria for discharge were absence of fever for at least 7 d, basically normal blood oxygen saturation without supplemental oxygen, substantial improvement in chest CT scans, and 2 consecutive throat-swab samples negative for SARS-CoV-2 RT-PCR test taken at least 24 h apart.

### Definitions

Fever was defined as axillary temperature of at least 37.3°C. Sepsis and septic shock were defined according to the 2016 Third International Consensus Definition for Sepsis and Septic Shock.^8^ Acute kidney injury (AKI) was diagnosed according to the Kidney Disease Improving Global Guidelines (KDIGO).^9^ ARDS was diagnosed according to the Berlin Definition.^10^ Acute cardiac injury was diagnosed if the serum levels of cardiac biomarkers (eg, cardiac troponin I) were above the 99th percentile upper reference limit or if new abnormalities were shown in electrocardiography and echocardiography.^7^ The illness severity of COVID-19 was defined according to the Chinese management guideline for COVID-19 (version 6.0).^11^ Coagulopathy was defined as a 3-s extension of prothrombin time or a 5-s extension of activated partial thromboplastin time. Hypoproteinemia was defined as blood albumin of less than 25 g/L. Exposure history was defined as exposure to people with confirmed SARS-CoV-2 infection or residence or travel history in Wuhan in the last month, especially in the last 2 wk. The worldwide accepted pneumonia severity scoring systems such as CURB-65.12] and Pneumonia Severity Index (PSI)^13^] were used to assess pneumonia severity. Critical illness evaluation systems, including Sequential Organ Failure Assessment (SOFA)^14^] and Acute Physiology and Chronic Health Evaluation II (APACHE II),^15^ were used to assess disease severity.

### Statistical Analysis

Categorical variables were presented as numbers (percentages) and analyzed using χ2 test or Fisher’s exact test. Continuous variables with skewed distribution were shown as median (interquartile ranges) and analyzed using Mann-Whitney U test. If the missingness of continuous variables did not exceed 30%, the median was used to fill in. The variables, including baseline characteristics, laboratory findings, were excluded those with more than 30% missing values and unconfirmed indicators (eg, exposure, which was self-reported). Logistic regression was used for univariable and multivariable analyses to explore the risk factors associated with in-hospital death. Variables with *P v*alue <0.1 entered into logistic multivariable analysis. Continuous variables were dichotomized. It was determined that whether or not with multicollinearity among independent variables. Forward-stepwise regression was used to select variables. Area under curve (AUC), sensitivity, and specificity were analyzed using the receiver operating characteristic curve (ROC). A nomogram was established based on the results of multivariable analysis. The C-index, decision curve, and clinical impact curve were used to verify the nomogram. A 2-sided *P* < 0.05 was considered statistically significant. All statistical analyses were performed using SPSS (version 25.0), R program (version 3.5.1), and Graphpad Prism (version 8.0).

## Results

### Demographics and Clinical Characteristics

**Figure 1.**
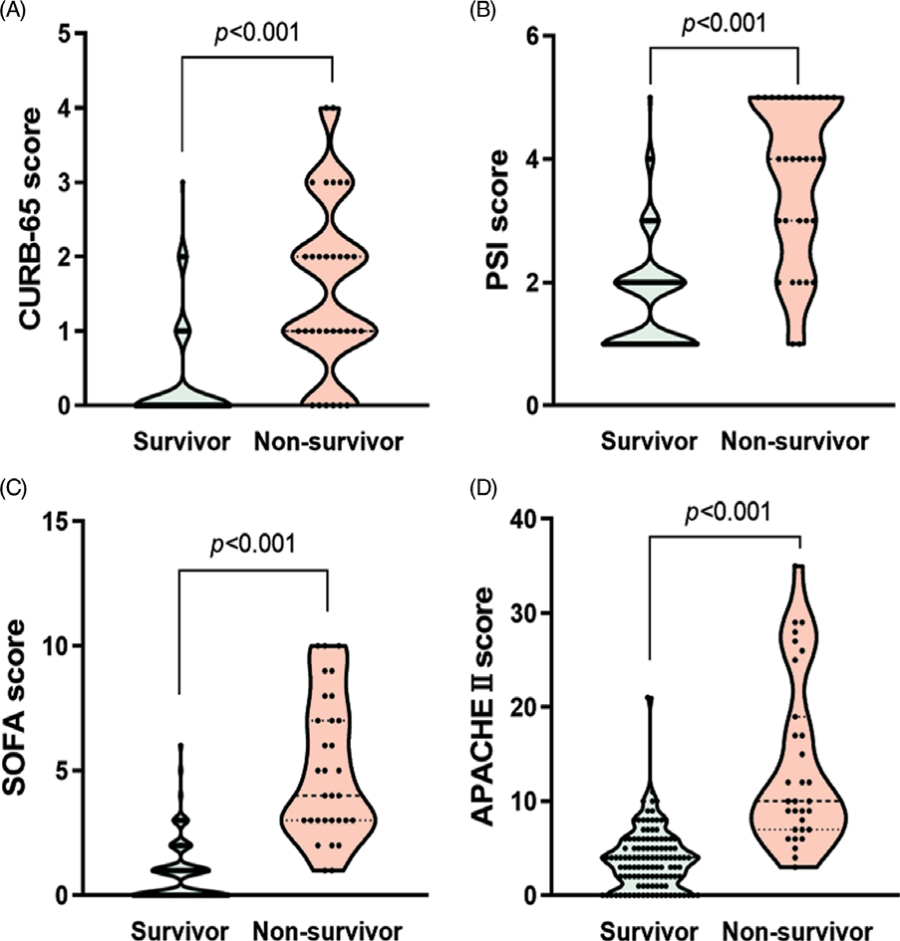
Comparison of pneumonia severity score and critical illness score between survivors and nonsurvivors. Violin diagram shows the higher CURB-65 score (A), PSI score (B), SOFA score (C), and APACHE II score (D) in the nonsurvivors. PSI, Pneumonia Severity Index; SOFA, Sequential Organ Failure Assessment; APACHE II, Acute Physiology and Chronic Health Evaluation II.

A total of 150 patients were enrolled in this study, including 31 dead patients and 119 discharged patients. As shown in Table 1, the nonsurvivors were older than the survivors (73 [IQR 62-79] y vs 48 [IQR 37-57]) y, *P* < 0.001). There were more male nonsurvivors (61.3%), while the survivors were dominated by females (58.0%). We noted that more nonsurvivors had hypertension, cardiovascular diseases, and cerebrovascular diseases than the survivors. The most common symptoms on admission were fever and cough, followed by fatigue. Dyspnea was more common in the nonsurvivors. Approximately one-third of the patients had a definite history of exposure before illness onset, and the median time from exposure to illness onset was 5.5 (IQR 4.8-10) d. General patients (83.2%) accounted for the majority in the survivor group, while severe and critical patients accounted for 93.5% in the nonsurvivor group. In addition, the nonsurvivors had higher CURB-65, PSI, SOFA, and APACHE II scores (Figure 1).

**Table 1.** Demographic and clinical characteristics of patients with COVID-19 on admission

	Total (*n* = 150)	Non-survivor (*n* = 31)	Survivor (*n* = 119)	*P* value
**Demographics**				
Age, y	52 (39.5-66.5)	73 (62-79)	48 (37-57)	<0.001
Sex, n (%)				
Male	69 (46.0%)	19 (61.3%)	50 (42.0%)	0.055
Female	81 (54.0%)	12 (38.7%)	69 (58.0%)	
Underlying diseases, *n* (%)	54 (36.0%)	23 (74.2%)	31 (26.1%)	<0.001
Hypertension	25 (16.7%)	14 (45.2%)	11 (9.2%)	<0.001
Diabetes	17 (11.3%)	11 (35.5%)	6 (5.0%)	<0.001
Cardiovascular disease	12 (8.0%)	9 (29.0%)	3 (2.5%)	<0.001
Cerebrovascular diseases	10 (6.7%)	7 (22.6%)	3 (2.5%)	<0.001
Chronic respiratory disease	6 (4.0%)	3 (9.7%)	3(2.5%)	0.195
Chronic kidney disease	2 (1.3%)	2 (6.5%)	0 (0.0%)	0.042
Malignancy	3 (2.0%)	2 (6.5%)	1 (0.8%)	0.109
Others	13 (8.7%)	3 (9.8%)	10 (8.4%)	1.000
Current smoker, *n* (%)	12 (8.0%)	2 (6.5%)	10 (8.4%)	1.000
Exposure history, *n* (%)	50 (33.3%)	8 (25.8%)	42 (35.3%)	0.318
**Clinical characteristics**				
Symptom, *n* (%)				
Fever (temperature ≥37·3°C)	115 (76.7%)	26 (83.9%)	89 (74.8%)	0.287
Cough	104 (69.3%)	22 (71.0%)	82 (68.9%)	0.825
Sputum production	49 (32.7%)	11 (35.5%)	38 (31.9%)	0.707
Chest discomfort	53 (35.3%)	15 (48.4%)	38 (31.9%)	0.088
Dyspnea	21 (14.0%)	11 (35.5%)	10 (8.4%)	<0.001
Fatigue	86 (57.3%)	27 (87.1%)	59 (49.6%)	<0.001
Myalgia	42 (28.0%)	6 (19.4%)	36 (30.3%)	0.229
Diarrhea	12 (8.0%)	2 (6.5%)	10 (8.4%)	1.000
Respiratory rate, breaths/min	20 (19-21)	21 (20-25)	20 (19-21)	0.016
Pulse, beats/min	80 (75-88)	86 (74-106)	80 (75-86)	0.134
Mean arterial pressure, mmHg	93 (89-98)	97 (90-108)	93 (88-97)	0.048
Disease severity status, *n* (%)				
General	101 (67.3%)	2 (6.5%)	99 (83.2%)	
Severe	40 (26.7%)	20 (64.5%)	20 (16.8%)	<0.001
Critical	9 (6.0%)	9 (29.0%)	0 (0.0%)	
CURB-65 score, *n* (%)				
0-1	126 (84.0%)	16 (51.6%)	110 (92.4%)	
2-3	22 (14.7%)	13 (41.9%)	9 (7.6%)	<0.001
4-5	2 (1.3%)	2 (6.5%)	0 (0.0%)	
PSI score, *n* (%)				
I	61 (40.7%)	2 (6.5%)	59 (49.6%)	
II-III	65 (43.3%)	10 (32.2%)	55 (46.2%)	<0.001
IV-V	24 (16.0%)	19 (61.3%)	5 (4.2%)	
SOFA score	1 (0-3)	4 (3-7)	1 (0-1)	<0.001
APACHE||score	4 (2-7.3)	10 (7-19)	4 (2-6)	<0.001
Time from exposure to illness onset, days*	5.5 (4.8-10)	6 (4.3-12.3)	5.5 (4.5-10)	0.975
Time from illness onset to hospital admission, days	7 (5-10)	7 (6-10)	7 (5-10)	0.286

Abbreviations: PSI, Pneumonia Severity Index; SOFA, Sequential Organ Failure Assessment; APACHE||, Acute Physiology and Chronic Health Evaluation.

* Data available for 30 patients, including 4 non-survivors and 26 survivors.

### Laboratory Findings and Imaging Features

Laboratory findings on hospital admission are summarized in Table 2. White blood cell counts and neutrophil counts were elevated, while lymphocyte counts and platelet counts were decreased in the nonsurvivor group compared with those in the survivor group. The nonsurvivors showed higher levels of total bilirubin, blood ureanitrogen (BUN), creatinine, fasting blood glucose, lactate dehydrogenase (LDH), and D-dimer, but lower albumin levels than the survivors. C-Reactive protein and procalcitonin (PCT) were also different between the nonsurvivors and survivors.

**Table 2.** Laboratory and radiographic findings of patients with COVID-19 on admission

	Total (*n* = 150)	Non-survivor (*n* = 31)	Survivor (*n* = 119)	*P-V*alue
**Laboratory findings**				
Hematologic				
White blood cell counts, × 10^9^ per L	5.7 (4.0-8.2)	9.1 (5.2-12.3)	5.5 (3.9-7.2)	<0.001
Lymphocyte counts, × 10^9^ per L	1.1 (0.7-1.6)	0.6 (0.4-0.8)	1.3 (0.9-1.7)	<0.001
Neutrophil counts, × 10^9^ per L	3.8 (2.3-6.2)	8.5 (4.1-10.9)	3.5 (2.1-5.2)	<0.001
Hemoglobin, g per L	124.5 (117.0-136.0)	128.0 (123.0-136.5)	123.0 (116.0-136.0)	0.298
Hematocrit, %	38.3 (35.6-41.9)	37.9 (35.6-41.8)	38.3 (35.6-42.2)	0.913
Platelet counts, × 10^9^ per L	191.5 (144.3-250.0)	121.0 (104.0-193.5)	198.0 (161.0-257.0)	<0.001
Biochemical				
Aspartate aminotransferase, U/L	19.4 (16.0-31.8)	31.0 (20.5-50.0)	18.0 (15.0-26.3)	<0.001
Alanine aminotransferase, U/L	19.0 (13.0-32.0)	24.0 (19.5-34.0)	17.0 (12.0-32.0)	0.044
Total bilirubin, μmol/L	11.4 (8.3-17.5)	19.3 (10.3-28.8)	10.6 (8.1-15.6)	<0.001
Albumin, g/L	37.8 (34.6-41.7)	33.3 (29.7-35.5)	39.1 (35.9-42.2)	<0.001
Globulin, g/L	27.8 (25.1-30.8)	30.3 (25.4-34.8)	27.4 (25.1-29.9)	0.018
Urea nitrogen, mmol/L	4.3 (3.2-6.2)	9.1 (4.6-13.3)	4.1 (3.1-5.0)	<0.001
Creatinine, μmol/L	73.5 (57.5-90.1)	88.7 (72.9-108.0)	70.2 (54.6-84.0)	0.001
Fasting blood glucose, mmol/L	5.8 (5.0-7.0)	7.8 (5.9-12.9)	5.8 (4.8-6.4)	<0.001
Lactate dehydrogenase, U/L	208.5 (178.3-270.8)	423.0 (208.5-571.0)	208.5 (168.5-230.0)	<0.001
Coagulation function				
Prothrombin time, s*	12 (11.4-12.7)	12.7 (12.0-13.6)	11.9 (11.1-12.5)	<0.001
Activated partial thromboplastin time, s*	31.2 (29.3-33.0)	31.0 (27.5-34.1)	31.2 (29.5-32.8)	0.595
D-dimer, μg/L*	196.0 (101.3-384.3)	638.0 (258.5-5189.5)	173.0 (88.5-264.0)	<0.001
Infection-related indices				
Erythrocyte sedimentation rate, mm/h^†^	29.0 (18.5-43.5)	29.0 (28.5-44.5)	29.0 (17.0-41.5)	0.165
C-Reactive protein, mg/L^‡^	9.2 (1.0-47.3)	57.6 (31.5-97.4)	7 (0.5-30.8)	<0.001
Procalcitonin, ng/mL, *n* (%)^§^				
<0.5	77 (83.7%)	8 (53.3%)	69 (89.6%)	
0.5-2	10 (10.9%)	5 (33.3%)	5 (6.5%)	0.007
>2	5 (5.4%)	2 (13.3%)	3 (3.9%)	
**Imaging features**				
Ground glass opacification, *n* (%)	129 (86.0%)	29 (93.5%)	100 (84.0%)	0.043
Pleural effusion, *n* (%)	10 (6.7%)	7 (22.6%)	3 (2.5%)	<0.001
Unilateral pulmonary infiltration, *n* (%)	26 (17.3%)	1 (3.2%)	25 (21.0%)	0.020
Bilateral pulmonary infiltration, *n* (%)	124 (82.7%)	30 (96.8%)	94 (79.0%)	0.020

* Data available for 141 patients, missing for 9 survivors.

† Data available for 111 patients, including 21 non-survivors and 90 survivors.

‡ Data available for 143 patients, including 25 non-survivors and 118 survivors.

§ Data available for 93 patients, including 15 non-survivors and 78 survivors.

**Figure 2.**
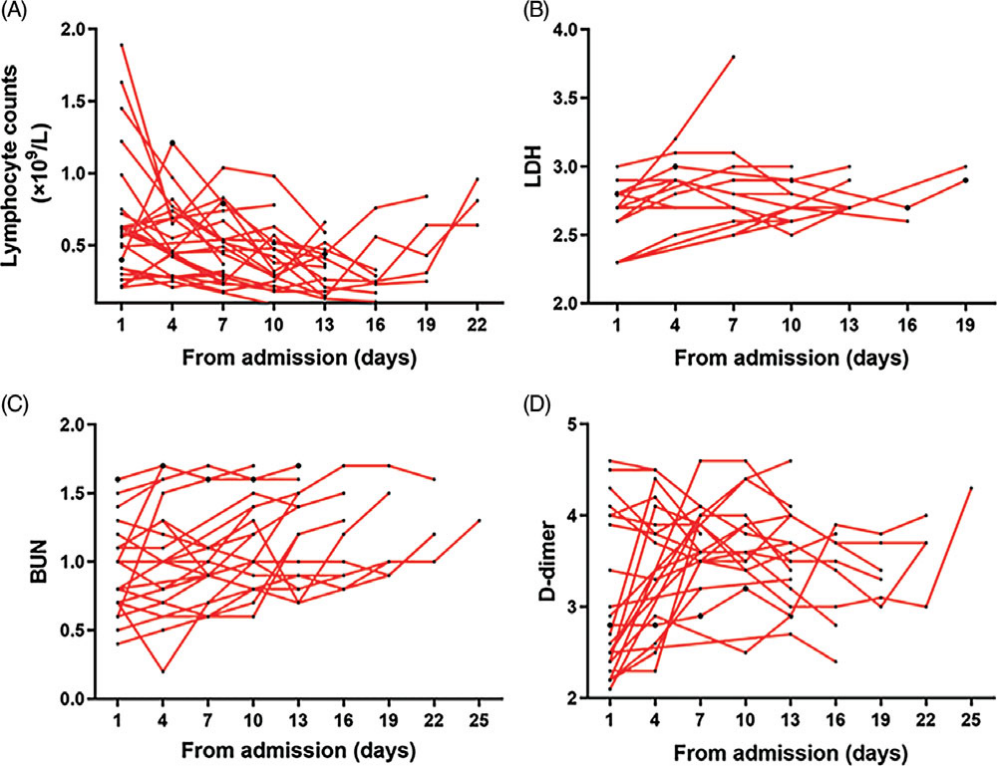
Temporal changes in laboratory markers from illness onset to death in nonsurvivors. (A) Line chart shows a dynamic decrease in lymphocyte counts after hospitalization. (B) LDH, (C) BUN, (D) D-dimer values basically show an upward trend throughout the clinical course. Lymphocyte counts, BUN, and D-dimer were obtained from 24 nonsurvivors. LDH was obtained from 15 nonsurvivors. LDH, BUN, and D-dimer values were log10-transformed for analysis, due to the wide range of variation. LDH, lactate dehydrogenase; BUN, blood urea nitrogen.

During the period from admission to death, laboratory indicators showed a dynamic change in the nonsurvivors, especially in the levels of lymphocyte counts, LDH, BUN, and D-dimer (Figure 2). The serial biomarker results were based on a very small subcohort because most patients were not continuously monitored during hospitalization.

The most common imaging features were ground glass opacification (86.0%) and bilateral pulmonary infiltration (82.7%). Pleural effusion (22.6% vs 2.5%, *P* < 0.001) occurred more frequently in the nonsurvivors.

### Clinical Outcomes

As shown in Table 3, of the 150 patients, sepsis was the most frequently observed complication (37.3%), followed by respiratory failure (26.7%). All of the nonsurvivors experienced sepsis. The common complications of the nonsurvivors included respiratory failure (93.5%), ARDS (83.9%), coagulopathy (77.4%), acidosis (64.5%), septic shock (61.3%), and AKI (61.3%). For the nonsurvivors, the median time from illness onset to respiratory failure was 9 (interquartile ration [IQR], 7-10) d; the median time from illness onset to ARDS was 10.5 (IQR 9-12) d; the median time from illness onset to septic shock was 18 (IQR, 14-23) d; the median time from illness onset to AKI was 16 (IQR, 10-22) d.

**Table 3.** Clinical outcomes

	Total (*n* = 150)	Non-survivor (*n* = 31)	Survivor (*n* = 119)	*P*-Value
Complication, n (%)				
Sepsis	56 (37.3%)	31 (100.0%)	25 (21.0%)	<0.001
Septic shock	19 (12.7%)	19 (61.3%)	0 (0.0%)	<0.001
Respiratory failure	40 (26.7%)	29 (93.5%)	11 (9.2%)	<0.001
ARDS	26 (17.3%)	26 (83.9%)	0 (0.0%)	<0.001
Acute cardiac injury	15 (10.0%)	15 (48.4%)	0 (0.0%)	<0.001
Heart failure	13 (8.7%)	12 (38.7%)	1 (0.8%)	<0.001
Acute kidney injury	21 (14.0%)	19 (61.3%)	2 (1.7%)	<0.001
Acidosis	20 (13.3%)	20 (64.5%)	0 (0.0%)	<0.001
Hypoproteinemia	20 (13.3%)	18 (58.1%)	2 (1.7%)	<0.001
Coagulopathy	26 (17.3%)	24 (77.4%)	2 (1.7%)	<0.001
Time from illness onset to respiratory failure, days	9 (6.3-11)	9 (7-10)	6 (3-17)	0.637
Time from illness onset to ARDS, days	–	10.5 (9-12)	–	–
Time from illness onset to septic shock, days	–	18 (14-23)	–	–
Time from illness onset to acute kidney injury, days	–	16 (10-22)	–	–
Time from illness onset to death or discharge, days	24 (18-30)	22 (13-25)	25 (19-30)	0.019
Hospital length of stay, days	16 (12-21)	13 (6-17)	17 (12-23)	0.002
Duration of viral shedding after COVID-19 onset, days	7 (4-10)	12 (7.8-15)	6 (4-10)	<0.001

Abbreviations: ARDS, acute respiratory distress syndrome.

The median time from illness onset to discharge was 25 (IQR, 19-30) d, whereas the median time to death was 22 (IQR 13-25) d. The median hospital durations of stay were 17 (IQR, 12-23) d for the survivors and 13 (IQR, 6-17) d for the nonsurvivors, respectively. The median duration of viral shedding of the nonsurvivors were longer than that of the survivors (12 [IQR, 7.8-15] d vs 6 [IQR, 4-10]) d; *P* < 0.001), even the virus was continuously detectable until death in 17 nonsurvivors.

### Risk Factors for In-hospital Death

On the univariable analysis, the risk factors associated with in-hospital death were age, hypertension, diabetes, and cardiovascular disease. The elevated respiratory rate, PSI score, SOFA score, APACHE II score, white blood cell counts, total bilirubin, BUN, creatinine, LDH, D-dimer, and the decreased white blood cell counts, lymphocyte counts, platelet counts, and albumin were also associated with in-hospital death (Table 4).

**Table 4.** Risk factors for in-hospital mortality

	Univariable analysis			Multivariable analysis		
	OR	95% CI	*P*-Value	OR	95% CI	*P*-Value
**Demographics**						
Age, years						
<65	1 (Ref)					
≥65	11.783	4.768—29.119	<0.001			
Underlying diseases						
Hypertension	8.806	3.156—20.716	<0.001			
Diabetes	10.358	3.439—31.196	<0.001	10.474	1.554—70.617	0.016
Cardiovascular disease	15.818	3.964—63.118	<0.001			
**Clinical characteristics**						
Respiratory rate, breaths per min						
≤24	1 (Ref)					
>24	5.000	1.783—14.021	0.002			
PSI score*	4.128	2.600—6.553	<0.001			
SOFA score*	3.212	2.079—4.961	<0.001	3.077	1.848—5.122	<0.001
APACHEII score*	1.502	1.268—1.779	<0.001			
**Laboratory findings**						
White blood cell counts, × 10^9^ per L						
4—10	1 (Ref)					
<4	0.081	0.026—0.248	<0.001			
>10	0.056	0.014—0.231	<0.001			
Lymphocyte counts, × 10^9^ per L						
≥0.8	1 (Ref)					
<0.8	12.676	5.011—32.068	<0.001			
Platelet counts, × 10^9^ per L						
≥100	1 (Ref)					
<100	6.900	1.812—26.273	0.005			
Total bilirubin, μmol/L						
≤20	1 (Ref)					
>20	8.359	3.321—21.042	<0.001			
Albumin, g/L						
≥35	1 (Ref)					
<35	7.589	3.195—18.025	<0.001			
Urea nitrogen, mmol/L						
<11	1 (Ref)					
≥11	18.413	4.672—72.560	<0.001			
Creatinine, μmol/L						
≤133	1 (Ref)					
>133	11.250	2.068—61.213	0.005			
Lactate dehydrogenase, U/L						
≤245	1 (Ref)			13.169	2.934—59.105	0.001
>245	22.927	7.918—66.384	<0.001			
D-dimer, μg/L						
≤500	1 (Ref)					
>500	9.107	3.700—22.417	<0.001			

Abbreviations: PSI, Pneumonia Severity Index; SOFA, Sequential Organ Failure Assessment; APACHE||, Acute Physiology and Chronic Health Evaluation||.

* Per 1 unit increase.

The multivariable logistic analysis indicated that higher SOFA score (odds ratio [OR], 3.077; 95% confidence interval [CI]: 1.848-5.122; *P* < 0.001), history of diabetes (OR, 10.474; 95% CI: 1.554-70.617; *P* = 0.016), and LDH greater than 245 U/L (OR, 13.169; 95% CI: 2.934-59.105; *P* = 0.001) were risk factors for in-hospital death (Table 4). The predictive equation for in-hospital death: logit(*P*) = −5.594 + 1.124 × SOFA + 2.578 × LDH ([LDH ≤ 245] = 0, [LDH > 245] = 1) + 2.349 × diabetes (without diabetes = 0, with diabetes = 1), *P* = e^logit(*P*)^ /1 + e^logit(*P*)^.

### Development and Validation of a Nomogram for Predicting In-hospital Death

**Figure 3.**
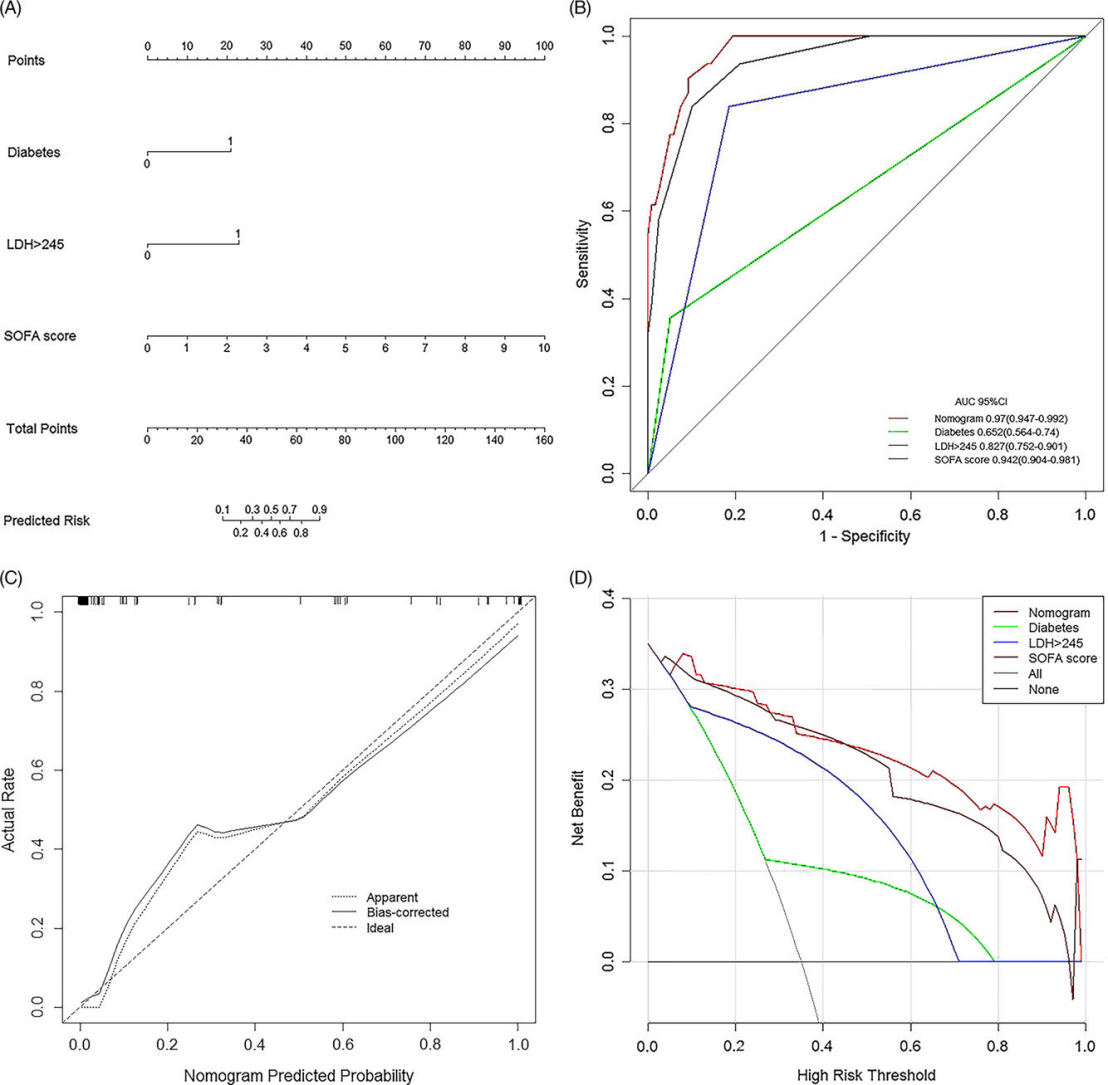
Prediction of in-hospital death of patients with COVID-19. A, Prognostic nomogram for predicting in-hospital death risk of patients with COVID-19. Prognostic patient’s value is located on each variable axis, and a line is drawn upward to determine the number of point nomogram for predicting in-hospital death risk of patients with COVID-2019. B, Area under the receiver operating characteristic curve (AUC) of SOFA score, diabetes, LDH, and the nomogram were 0.942, 0.827, 0.652, and 0.970, respectively. Calibration curve (C) and clinical impact curve of the nomogram (D), in which the predicted probability of in-hospital death was highly consistent with the actual observation and had good net benefit.

The independent predictors were used to establish a nomogram, and the points of each variable are shown in Figure 3A. The ROC curve analysis revealed that the AUC of the nomogram was 0.970 (95% CI: 0.947-0.992), showing good accuracy in predicting the risk of in-hospital death (Figure 3B). The sensitivity was 90.3%, and the specificity was 90.8%. The favorable calibration curve indicated that the prediction by the nomogram was highly consistent with the actual observation (Figure 3C). Moreover, the clinical impact curve (Figure 3D) indicated that the nomogram had good net benefits for the identification of the fatal outcome of patients with COVID-19.

Fifty-two of 512 hospitalized patients with COVID-19 between March 5, 2020, and May 1, 2020, including all 12 dead patients and 40 discharged patients who were randomly selected according to hospitalization number, were enrolled to perform an external validation. The AUC of the nomogram was 0.923 (95% CI: 0.828-1.000), and the predictive accuracy was 0.942. The model was confirmed to be reliable.

## Discussion

Most of patients with COVID-19 present with mild flu-like symptoms and recover quickly. However, many severe patients show rapid progression and develop multiple organ dysfunction, even death, indicating that recognition of risk factors are essential to identify those potentially needing critical care and management at an early stage. Several studies focused on risk factors associated with poor prognosis, including elder age, comorbidities, lymphopenia, D-dimer greater than 1 μg/L, elevated CRP, high LDH, high hypersensitive troponin I, high interleukin-6, hyperglycemia, and hypoproteinemia. However, these clinical and laboratory indicators are independent,^2,5,16-18^ which cannot comprehensively reflect the prognosis of COVID-19.

To our knowledge, no mature and reliable scoring system has been established to assess the risk of death in hospital with COVID-19. SOFA score is a low-cost, 2-min bedside clinical tool introduced to facilitate early recognition of sepsis and multi-organ dysfunction. SOFA score is a consistent and convincing tool that can be used across different patient populations and clinical settings. In the current study, we determined that sepsis occurred in 37.3% of patients with COVID-19 due to viral infection. We also identified SOFA score as an independent risk factor for death in adults who were hospitalized due to COVID-19.

Reports from the Chinese Center for Disease Control and Prevention from 44,672 confirmed cases of COVID-19 showed that patients with diabetes had a 3-fold higher overall case-fatality rate than those without diabetes (7.3% vs 2.3%, respectively).^19^ According to our results, we believed that increasing odds of death in hospital was associated with history of diabetes. The elevated LDH was confirmed as a predictive biomarker of death, consistent with those in previous reports.^16,20^ Furthermore, our study developed a nomogram model based on risk factors selected by multivariable logistic regression analysis to accurately predict fatal outcomes of patients with COVID-19. The nomogram model performed well in predicting in-hospital death, supported by external validation.

Few studies reported the long shedding of SARS-CoV-2 RNA, especially in severe patients.^2,21^ Among male patients, delayed admission to hospital after illness onset, and invasive mechanical ventilation were associated with prolonged SARS-CoV-2 RNA shedding.^22^ In the current study, we found that the duration of SARS-CoV-2 viral shedding had median values of 6 d in the survivor group and 12 d in the nonsurvivor group; 17 patients were nucleic acid positive until death. Although most studies have used qualitative or quantitative PCR tests as a diagnostic marker for infectious SARS-CoV-2, caution is required when applying such data to assess the duration of viral shedding and infection potential because PCR does not distinguish between infectious virus and noninfectious nucleic acid.

COVID-19 mainly injures the respiratory system, and some patients rapidly progress to ARDS. We found that the incidence of ARDS was 17.3%, and the median time from illness onset to ARDS was 10.5 (IQR, 9-12) d, consistent with previous reports.^2,23^ At present, the mechanisms of COVID-19-related ARDS remain unclear. The entry of pathogenic SARS-CoV-2 in humans leads to the activation of inflammatory cells, specifically CD4 lymphocytes, which subsequently transform into T helper 1 (Th1) cells. Activated inflammatory cells (Th1 cells and macrophages) enter the pulmonary circulation and induce cytokines (ie, “cytokine storm”), which lead to rapid and wide-spread damage of the pulmonary epithelium and alveolar cells.

A recent study of the single-cell transcriptome analysis found that ACE2 genes were significantly expressed in podocytes and proximal convoluted tubules as potential hosts cells targeted by SARS-CoV-2; this work suggests that the kidney might be an important target organ for SARS-CoV-2.^24^ A retrospective cohort study reported that mortality was higher in COVID-19 with AKI versus COVID-19 patients without AKI (60.5% vs 27.4%), and AKI was an independent predictor of mortality.^25^ Our study revealed that AKI incidence rates of 14.0% in all hospitalized patients and 61.3% in the nonsurvivors. These data provide robust evidence to support that patients with COVID-19 should be closely monitored for the development of AKI and measures taken to prevent it.

This study has several limitations. First, our study was conducted in 1 hospital, thereby potentially limiting the generalizability to hospital settings, especially in terms of the demographic characteristics of the patient population and the external validation of the prediction models. Second, missing data on some variables, such as erythrocyte sedimentation rate (ESR), PCT, and cardiac troponin I, may cause bias in the estimation and reduce the representativeness of the samples. Finally, interpretation of our findings might be limited by the small sample size.

In conclusion, we found that high SOFA score, history of diabetes, and LDH greater than 245 U/L were risk factors for in-hospital death of adult patients with COVID-19. The nomogram proposed in our study objectively predicted the prognosis of patients with COVID-19.
